# The Melamine Incident: Implications for International Food and Feed Safety

**DOI:** 10.1289/ehp.0900949

**Published:** 2009-08-06

**Authors:** Céline Marie-Elise Gossner, Jørgen Schlundt, Peter Ben Embarek, Susan Hird, Danilo Lo-Fo-Wong, Jose Javier Ocampo Beltran, Keng Ngee Teoh, Angelika Tritscher

**Affiliations:** World Health Organization, Department of Food Safety and Zoonoses, Geneva, Switzerland

**Keywords:** food safety, infant formula, kidney stones, melamine, milk powder, renal failure, risk assessment, World Health Organization

## Abstract

**Background:**

A major food safety incident in China was made public in September 2008. Kidney and urinary tract effects, including kidney stones, affected about 300,000 Chinese infants and young children, with six reported deaths. Melamine had been deliberately added at milk-collecting stations to diluted raw milk ostensibly to boost its protein content. Subsequently, melamine has been detected in many milk and milk-containing products, as well as other food and feed products, which were also exported to many countries worldwide.

**Objectives:**

The melamine event represents one of the largest deliberate food contamination incidents. We provide a description and analysis of this event to determine the global implications on food and feed safety.

**Discussions:**

A series of factors, including the intentional character of the milk contamination, the young age of the population affected, the large number of potentially contaminated products, the global distribution of these products, and the delay in reporting led this event to take on unexpected proportions. This incident illustrated the complexity of international trade of food products and food ingredients that required immediate actions at international level.

**Conclusion:**

Managing food-safety events should be done internationally and early on as soon as multinational consequences are expected. Collaboration between food-safety authorities worldwide is needed to efficiently exchange information and to enable tracking and recalling of affected products to ensure food safety and to protect public health.

An increased incidence of kidney stones and renal failure among infants has been publicly reported in China from early September 2008 onward. The source of the illness was traced to the contamination of infant formula with melamine. Investigations showed that melamine had been deliberately added to diluted raw milk to boost its apparent protein content. Previous outbreaks of renal failure related to melamine, a molecule high in nitrogen content, have been reported in pets in 2004 in the Republic of Korea and in 2007 in the United States when the substance was added deliberately to a pet food ingredient (Brown et al. 2007). Commonly used methods for protein analysis do not distinguish between nitrogen from protein and from nonprotein sources. Thus, the addition of melamine can lead to an incorrectly high protein reading. Because melamine is cheap and easily accessible, there is an economic incentive for its (illegal) addition.

Melamine is listed by the Organisation for Economic Co-operation and Development (2009) as a high production volume chemical. The main use of melamine is in the synthesis of melamine formaldehyde resins for manufacture of laminates, plastics, coatings, commercial filters, glues and adhesives, and some dishes and kitchenware.

After acknowledging this food-contamination incident, direct communication between the Chinese Ministry of Health and the World Health Organization (WHO) led to information sharing through the WHO/Food and Agricultural Organization International Food Safety Authorities Network (INFOSAN) to countries worldwide.

## Unfolding of the Global Event

On 9 September 2008, the *Shanghai Daily* reported that 14 infants from Gansu Province were suffering from kidney stones after drinking a particular brand of powdered infant milk formula (ProMED-mail 2008a). Although the exact onset date of illness resulting from contaminated infant formula and the beginning of the contamination itself remain unknown, it appears that companies received customer complaints about sick babies with discolored urine as early as December 2007 and that the first child died 1 May 2008 (Lancet 2009). Up until 12 September 2008, the State Council of China reported 432 cases and 1 death. All the infants identified with kidney stones had consumed infant formula produced by the Sanlu Group.

Subsequently, Chinese authorities announced the seizure of > 2,000 tons of milk powder from a Sanlu warehouse and the recall of about 9,000 tons of milk powder. A wide investigation into the extent of melamine contamination of dairy products revealed that 22 manufacturers of powdered infant formula were selling melamine-contaminated products. In the Sanlu products, melamine levels were reported to be as high as 2,563 mg/kg (ProMED-mail 2008b). Two other producers of powdered infant formula reported exports to Bangladesh, Burundi, Gabon, Myanmar, and Yemen. The Agri-Food and Veterinary Authority of Singapore (2008) reported the first melamine findings outside of China in Chinese milk and milk products on 17 September 2008; on 20 September, the government of Hong Kong Special Administrative Region announced that a 3-year-old girl developed kidney stones after consuming contaminated milk (Centre for Health Protection 2008).

Soon after, melamine was found in liquid milk and yogurts, frozen deserts, powdered milk and cereal products, confectionaries, cakes and biscuits, protein powders, and some processed foodstuffs. Subsequently, a variety of nondairy products originating from China were found to be contaminated with melamine. These products included ammonium bicarbonate [Rapid Alert System for Food and Feed (RASFF) 2008c], animal feed and animal feed ingredients (RASFF 2008b, 2008c), dried whole egg, fresh hen eggs (Centre for Food Safety 2008), and nondairy creamer (Korean Food and Drug Administration 2008).

## Levels of Contamination and Types of Products Affected

Over the course of the incident, different national authorities conducted laboratory tests for melamine. WHO collated analytical results obtained either from data published on official government web sites or through direct contact with national authorities via the INFOSAN network. It should be noted that not all national authorities that tested for melamine published their results or reported them to WHO; some authorities did not report positive results if the levels of contamination were below the regulatory or action limits for melamine in the country, which is most commonly 1.0 mg/kg for infant foods and 2.5 mg/kg for other food products. Hence, the data on product contamination presented here are not fully representative of the total number of products that might have been tested. It should also be noted that the results reported by the various laboratories were obtained using different analytical methods with varying limits of detection and quantification. Keeping these limitations in mind, we present the most complete collection of global data from the 2 October 2008 event to 31 January 2009 in Tables 1 and 2. Table 1 summarizes 326 individual analytical results for melamine in a variety of food products: an analysis of dairy products conducted by China’s General Administration of Quality Supervision, Inspection and Quarantine as of 2 October 2008 (*n* = 77), and the results reported by other national food-safety authorities (*n* = 249), as published on official web sites or reported directly to WHO via INFOSAN (up to 31 January 2009). Table 2 shows the total number of positive results, reported as individual results as summarized in Table 1, plus 74 results reported as ranges rather than as individual results. Overall, these results illustrate the broad range of melamine levels found in the different product categories.

## Health Impact

As the incident developed, updates on affected infants and children were provided by the Chinese Ministry of Health. The most recent update confirmed a total of 6 deaths and 294,000 cases associated with the consumption of melamine-contaminated milk and milk products as of 1 December 2008 (Chinese Ministry of Health 2008) (Table 3).

Two clinical case reports for a total of 74 children who were hospitalized with either acute renal failure or confirmed renal stones state the average duration of hospitalization was 13–16 days (WHO 2009). Of the 2,085 children screened, 17% (348 children) had stones, but only 25% had symptoms. In another study involving 589 children, 8.5% had stones, 19% were suspected of having stones, and 72.5% had no stones; all children were equally likely to have symptoms (Guan et al. 2009). This finding suggests that there may be many more children with urinary tract calculi or stones as a consequence of consuming melamine-contaminated products who remain asymptomatic and therefore undiagnosed. Urinary tract calculi or stones were found in kidney, ureter, or bladder. A study of children with and without urinary tract calculi in Hangzhou, China, found risk factors for calculi included long duration of formula feeding, high melamine content in infant formula, and minimal water intake (Zhang et al. 2009). The median duration of consumption of contaminated formula reported by one study of 25 children diagnosed with calculi was 8 months (range, 15 days to 13 months). In another study, 40 Sanlu milk powders collected at random in children’s homes were analyzed: 93% contained melamine and 73% also contained the melamine analog cyanuric acid. Melamine concentrations ranged from 150 mg/kg to 4,700 mg/kg (median, 1,900 mg/kg), and cyanuric acid concentrations ranged from 0.4 to 6.3 mg/kg (median, 1.2 mg/kg) (WHO 2009).

In a screening study conducted in Taiwan, China, Wang et al. (2009) reported the mean duration of exposure as 7.19 months (range, 0.67–36 months) in the high-exposure group, versus 17.4 months (range, 3–48 months) in the low-exposure group (defined as children who had consumed brands of contaminated milk with lower measured melamine levels).

## Countries Affected and Regulatory Response

**Figure 1:**
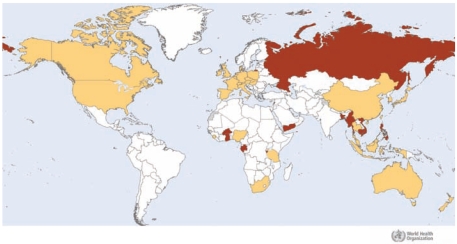
Global distribution of melamine-contaminated products as reported to INFOSAN and published on national official web sites. Light shading indicates countries that reported melamine findings in products originating from China or in products containing ingredients from China. The positive results were transmitted to WHO directly by the country, or by another relevant authority, or via the country’s official web site (Australia, Austria, Belgium, Canada, China, Hong Kong, Macao, Taiwan, Czech Republic, Denmark, France, Germany, Hungary, Indonesia, Ireland, Italy, Japan, Malaysia, Malta, Netherlands, New Zealand, Nigeria, Poland, Republic of Korea, Singapore, Slovakia, Slovenia, Solomon Islands, South Africa, Spain, Switzerland, Thailand, United Kingdom, Tanzania, and United States). Dark shading indicates countries to which import of contaminated products occurred, as declared by the exporting country, and countries that reported the import of contaminated products (Bangladesh, Brunei, Burkina Faso, Burundi, Cambodia, Gabon, Ghana, Lebanon, Myanmar, Palau, Philippines, Russian Federation, Seychelles, Viet Nam, Yemen). Data from WHO Map Production, by public health information and geographic health information systems, WHO 2009; all rights reserved. The boundaries and names shown and the designations used on this map do not imply the expression of any opinion whatsoever on the part of WHO concerning the legal status of any country, territory, city, or area or of its authorities, or concerning the delimitation of its frontiers or boundaries. Dotted lines on maps represent approximate border lines for which there may not yet be full agreement.

In total, 47 countries (Figure 1) received melamine-contaminated products, as reported to INFOSAN or published on each country’s official government web site, either through direct import or through third countries. Illegal distribution of contaminated product from China has also been demonstrated (RASFF 2008a).

Countries responded through a wide range of actions—from no action at all to the ban of all imports of milk and milk products from China. A number of countries implemented testing of all imported Chinese products. Other countries focused testing on implicated products, and 68 countries banned or recalled foods suspected of containing melamine (Bhalla et al. 2009). Several countries established (interim) limits for melamine in food and feed (e.g., Australia, Canada, China, European Union, Malaysia, New Zealand, United States). Other countries took the approach that melamine should be absent (i.e., a “zero-tolerance” approach). However, low levels (usually in the microgram per kilogram range) of melamine are found in some foods, not as a result of adulteration but through normal food production and processing (e.g., migration from food contact material, pesticides, or fertilizer use). Such levels are not a health concern. Moreover, “zero tolerance” in practice is dependant on the performance of the analytical method used, hence the actual acceptable level varies.

To respond to the request for information from countries around the word, INFOSAN elaborated and disseminated through its network lists of contaminated products, a list of laboratories that could analyze for melamine, a list of analytical methods for melamine and cyanuric acid in food and feed, and a list of limits set by national food safety authorities.

## Toxicology and Risk Assessment

Several national and regional authorities around the world and the WHO have issued preliminary risk assessments and guidance on levels in food, mainly based on information from the 2007 pet-food incident, as a first pragmatic approach for public health protection (WHO 2008). Subsequently, a meeting of independent international scientific experts was organized by WHO and the following brief summary provides relevant aspects of the report (WHO 2009).

### Toxicology

#### Absorption, distribution, metabolism, and excretion

Melamine and its structural analog cyanuric acid are rapidly absorbed and rapidly excreted almost completely unmetabolized in the urine. The elimination half-lives for these two compounds are about 3 hr (WHO 2004, 2009). No information is available for other structural analogs, and no information is available on the absorption and excretion of the melamine-cyanurate complex.

#### Toxicity of melamine

Melamine is of low acute toxicity, with oral median lethal doses in mice and rats exceeding 3,000 mg/kg body weight. Several subchronic studies in rats and mice are reported [Heck and Tyl 1985; National Toxicology Program (NTP) 1983] at doses up to 18,000 mg melamine/kg feed. The main observed toxicity was related to the excretory organs, kidney, and bladder. The most consistent and dose-related effects observed were bladder-stone formation and hyperplasia of the bladder epithelium. These effects were more pronounced in males than in females.

Melamine has been tested for carcinogenicity by oral administration in mice and rats (NTP 1983). The most significant and consistent finding from these studies was the production of urinary bladder carcinomas in male rats. In male mice, urinary bladder hyperplasia was detected. All tumor findings correlated strictly with calculus formation and exposure to high doses. In female rats, chronic inflammation of the kidney, distinct from chronic nephropathy in aging rats, was observed.

Melamine is not genotoxic, and it is not considered a reproductive and developmental toxicant.

#### Toxicity of cyanuric acid

The toxicologic profile of cyanuric acid is very similar to that of melamine. It is of low acute oral toxicity, and subchronic toxicity at concentrations up to 5,375 mg/L in drinking water resulted in a low incidence of bladder calculi at the highest dose. In a 2-year study of rats that were given sodium cyanurate in the drinking water at doses estimated up to 371 mg/kg body weight per day (5,375 mg/L), no substance-related increase in tumor incidence was observed (WHO 2004).

Cyanuric acid is not considered to be genotoxic, and it is not teratogenic or a reproductive toxicant.

#### Combined toxicity

From previous incidents in pets and livestock, and after experimental studies to investigate the combined effects of melamine plus cyanuric acid in cats (Puschner et al. 2007) and in fish and pigs (Reimschuessel et al. 2008), it is apparent that oral exposure to melamine given simultaneously with cyanuric acid caused much more severe renal damage than did oral exposure to melamine or cyanuric acid alone.

### Risk assessment

The formation of bladder calculi was identified as the most relevant end point, and because the calculi formation is dose-dependent or local-concentration dependant, with no signs of significant accumulation, the subchronic studies in rats serve as the basis for the risk assessment.

By applying dose–response modeling to the combined data for male rats from the two subchronic feeding studies, a 95% lower bound of the 10% benchmark dose (BMDL_10_) of 415 mg/kg diet was calculated. Dietary conversion and an additional feed-intake–reduction factor of 14% led to the BMDL_10_ of 35 mg/kg body weight per day. An uncertainty factor of 200 was applied, deriving a tolerable daily intake (TDI) of 0.2 mg/kg body weight (rounded to one significant figure). The uncertainty factor comprises the default 100-fold factor plus an extra 2-fold factor to account for sensitivity of infants and for data uncertainty in relation to possible underreporting of bladder calculi due to tissue preparation.

For adults, estimated exposure from “baseline” levels of melamine, defined as levels in food that do not result from adulteration or misuse from all sources, has been estimated to be up to 13 μg/kg body weight per day. Conservative exposure estimates from adulterated products from this incident were 0.8–3.5 times the TDI. For comparison, estimated exposure of infants in China to adulterated infant formula, at median levels of the most affected brand, ranged from 8.6 to 23.4 mg/kg body weight per day. These levels are about 40–120 times the TDI and explain the dramatic health outcome in Chinese infants.

## Discussion

### Sources and levels of contamination

The sources of melamine contamination have been divided into “baseline” levels, which refer to levels in food that do not result from adulteration or misuse, and “adulteration” levels, which refer to levels in food that result from the intentional illegal addition of melamine to food or feed (WHO 2009). Such a distinction is useful for practical purposes, but it is evident that a clear distinction is not always possible. For example, low levels of melamine in food could result from carryover from adulterated animal feed.

Baseline concentrations of melamine are present in the environment and in the food chain as a result of the widespread use of materials that contain melamine. Generally, baseline levels are expected to be < 1 mg/kg (WHO 2009), and these levels are not considered to be a health concern. Melamine concentrations in food and animal feed above baseline levels are considered to be the result of misuse or adulteration. The high levels of melamine detected in many products related to the 2008 incident are a clear indication of adulteration.

Data showing the presence of melamine in animal tissue (including fish), milk, and eggs demonstrate that carryover from feed to tissues, milk, and eggs does occur.

**Figure 2:**
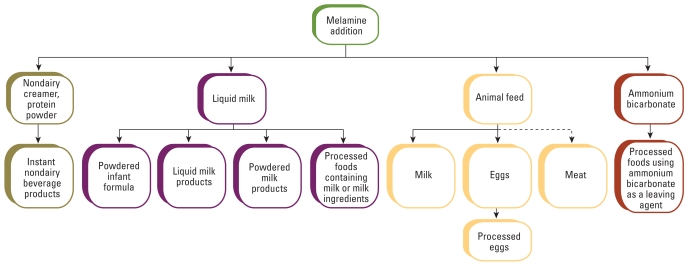
Flow chart of the melamine-contamination chain from adulteration. Solid lines indicate contaminated products as observed during the 2008 incident. Dashed lines indicate possible contamination but not reported during the 2008 incident.

Figure 2 presents the four major possible paths for deliberate contamination: *a*) liquid milk in the milk-collecting stations that was then used in the production of powdered infant formula, liquid and powdered milk products, and processed milk-containing foods; *b*) animal feed that resulted in contamination of milk, eggs, and potentially meat; *c*) nondairy creamer and protein powder that lead to the contamination of instant nondairy beverage products; and *d*) ammonium bicarbonate that was used to produce several types of processed food.

From the wide-ranging levels of melamine found in the different product categories (Table 2), it cannot be immediately determined which of the positive results are due to baseline contamination and which are due to intentional misuse and adulteration of melamine in food and feed. It is currently still unclear how products such as ammonium bicarbonate, nondairy creamer, and protein powder have been contaminated with melamine. However, it is assumed that melamine, of high purity grade as white powder, was added directly to such products, as it would serve as an inexpensive substitute for other raw material or boost apparent protein content.

The detection of melamine in products such as dried egg powder and whole eggs indicates carryover of melamine from feed to food products. In one particular incident in South Africa, it was reported that locally produced powdered-milk products were contaminated with melamine due to the use of old stocks of contaminated feed, from the 2007 incident, for dairy cattle (Animal Feed Manufacturers Association 2008). The discovery of very high levels of melamine (21,000 mg/kg) (RASFF 2008c) in rice-protein concentrate from China, an animal feed ingredient, would also seem to serve as corroborating evidence that the risk of carryover from feed to food products is likely. Further studies on this subject are warranted.

Overall reports of > 400 positive results in a broad variety of food products have been compiled by WHO up to 31 January 2009. It is assumed that more analytical results are available worldwide and that monitoring is ongoing. It is interesting to note that almost no positive results for melamine in infant formula have been reported by authorities outside of China. This result may be due to rapid actions such as import bans. However, many countries require premarket approval or notification for infant formula, and many countries may not allow Chinese infant formula in their market. In Canada, one study was performed by authorities to study baseline levels in local infant formula, where melamine was detected in 60 of the 80 local products sampled, at concentrations ranging from 4.31 to 346 μg/kg (Health Canada 2008). It served to identify baseline levels in infant formula.

### Health impact

Because the epidemiologic studies showed that many children with identified calculi where asymptomatic, it is likely that there were many more cases that were not brought to the attention of medical authorities. Considering the global distribution of affected products, as well as informal distributions, it is also possible that there are unidentified and therefore unreported cases in countries other than China. Also, many children had small calculi or stones that were not detectable with standard methods, further leading to a possible underreporting of affected children.

There is a lack of information on the long-term effects of melamine in humans, which makes predicting the subchronic and chronic health problems that might follow from the 2008 incident difficult. The carcinogenic effects reported in animal studies subsequent to irritation caused by stones formed after high exposure (International Agency for Research on Cancer 1999) do raise health concerns. Thus, it is essential that treatment to eliminate calculi and stones be continued and that long-term studies into the human health effects of melamine be carried out. Information obtained from the current incident is critical to identify the long-term effects of high levels of melamine consumption in humans including conducting large-scale epidemiologic investigations such as longitudinal cohort studies, long-term follow-up of affected cohorts, and more extensive case findings to establish the total population affected.

### Toxicology

The TDI is derived from short-term toxicology studies in animals. These studies were not designed to investigate bladder or kidney crystal and calculi formation. However, they form a strong basis for risk assessment, particularly considering that the effects seen in humans are similar to the effects seen in animals. Nevertheless, uncertainty remains, particularly in light of the new findings that melamine crystals dissolve rapidly in formalin, which is routinely used for tissue fixation. This may have led to an underestimation of the formation of bladder crystals, the critical effect used as a basis for derivation of the TDI.

It is important to note that the adverse effects seen in experimental animals, and probably in humans as well, are due to a local physical effect rather than a systemic effect. Melamine and its analogs are rapidly excreted, and crystal formation occurs only at high doses when a critical concentration is reached in the excretory organ. It appears that only when this threshold is exceeded do adverse effects occur. However, available data do not allow identification of this threshold concentration, for melamine alone or in combination with its analogs, and further investigations are needed.

### Delay of reporting

As soon as WHO requested information, the Chinese authorities acknowledged the melamine event and cooperated with WHO. According to information provided to INFOSAN by Chinese authorities on 29 September 2008, parents of the infants who consumed Sanlu formula filed complaints to the company as early as December 2007, and the company had detected melamine in its products in June 2008. The company only reported its findings to the local government in August 2008, followed by a further delay until 9 September when the incident was reported to the provincial government.

A timely response is important to assure appropriate actions to limit the spreading of contaminated product and to ensure that all contaminated product—domestic and exported—can be taken off the market. There is a clear responsibility for all parties involved in the food-production chain (producers and authorities), to release immediately any information related to any contamination with a possible human health impact. Withholding such information will negatively affect health outcome and the credibility of all involved.

### International consequences and lessons learned

The managing of outbreaks depends strongly on a well-structured food-safety system; communication and access to information are key components that will determine a positive reaction to an incident. China, the country at the origin of this incident, shared information internationally through INFOSAN. Data from outside China were collated and also communicated to national authorities through the INFOSAN network. This incident, and the rapid spread of the affected products worldwide, has evidenced the need for a mechanism for coordination and information exchange linking food-safety authorities and promoting the rapid exchange of information.

In any important food-safety event, there is an urgent need to provide the best available scientific knowledge in the area. Such knowledge can save lives and help to control an outbreak. Sharing information across borders is essential to obtain the best advice possible and to avoid confusion when tackling international events. This information should include international agreement about testing and reporting methodology; without these, coherent international analysis and action are not possible. In general, there is a need to achieve international scientific agreement relative to the risk of melamine in food and feed. A WHO expert meeting (WHO 2009) provided the first international forum for exchange and joint analysis of data in this area. The Chinese authorities should be commended for the pertinent data provided at this meeting.

During the 2008 incident, significant confusion existed regarding which level of melamine in food presents a human health risk. Communication relative to the levels protective of health was made additionally difficult by the fact that countries in some cases published different “action” levels; some countries even used any melamine concentration detected as a signal for action. It is important for authorities to present clear, understandable reasoning for any action or nonaction taken. There is a need for a common understanding of the underlying science that ideally leads to one harmonized international set of limits in food and feed, typically achieved through the Codex Alimentarius Commission.

### Ensuring food safety to protect public health

Some 68 countries have taken different restrictive trade measures against a range of food products originating from China. These were introduced at the onset of the event in September 2008. Several months later, questions still remain as to when and how to consider this incident under control. For many trading partner countries, it is difficult to assess the safety of future supplies and to decide on what basis to lift the restrictive measures imposed. For the Chinese authorities, demonstrating that the measures they have put in place will ensure an appropriate level of confidence in the future safety of food products is also posing a challenge.

This incident has clearly demonstrated a need to develop, at the international level, risk-based import-inspection systems. In addition, guidance regarding the necessary measures to demonstrate, with a certain degree of confidence, the appropriate levels of safety when a major food-safety event has seriously shaken confidence in the capabilities of a system to ensure the safety of the food it produces.

This incident has also clearly demonstrated that food safety can only be ensured if all the stakeholders along the food chain are sharing information and data in a timely manner.

## Figures and Tables

**Table 1:** Products positive for melamine.

Product category	No. of positive products	No. of samples with different levels of melamine (mg/kg)					
		< 1	1 to ≤ 2.5	2.5 to ≤ 10	10 to ≤ 100	100 to ≤ 1,000	> 1,000
Reported by Chinese authorities							
Powdered infant formula	22	2	1	2	13	3	1
Liquid milk and yogurt	24	4	5	15	0	0	0
Powdered milk products	31	0	1	2	7	10	11
Total	77	6	7	19	20	13	12

Reported by other national authorities^a^							
Liquid milk and yogurt	17	0	4	7	5	1	0
Powdered milk products	23	14	3	2	3	1	0
Frozen dairy products	5	0	0	2	3	0	0
Confectionary products^b^	158	0	4	92	53	9	0
Snack foods^c^	13	1	1	5	6	0	0
Frozen processed foods^d^	15	5	3	2	5	0	0
Ammonium bicarbonate	2	0	0	0	0	2	0
Nondairy creamer	1	0	1	0	0	0	0
Protein powder	2	0	0	2	0	0	0
Dried egg powder and liquid eggs	5	3	1	1	0	0	0
Whole eggs	4	0	0	4	0	0	0
Animal feed	4	0	0	2	1	0	1
Total	249	23	17	119	76	13	1

a Does not include results that were reported as a range rather than as a single data point, because the number of samples taken for these results was not apparent.

b Includes products where the use of milk as an ingredient was apparent, such as in chocolate or biscuits with cream filling.

c Includes products where the use of milk as an ingredient was not obvious, such as in potato crackers or rice crisps.

d Includes products such as frozen pizza dough or frozen fried chicken.

**Table 2:** Range of melamine levels detected in various food products.

Product category	Contamination range (mg/kg)	No. of positive products
Powdered infant formula	0.1–2,563	22
Liquid milk and yogurt	0.6–648	52
Powdered milk products	< 1–6,196	56
Frozen dairy products	4.4–60.8	6
Confectionary products	0.3–945.9	200
Snack foods	0.5–54	17
Frozen processed foods	0.5–41	20
Ammonium bicarbonate	33.4–508	4
Nondairy creamer	1.5–6,694	2
Protein powder	3.8–8.3	2
Dried egg powders and liquid eggs	0.1–5	8
Whole eggs	2.9–4.7	4
Animal feed	3.3–21,000	7

**Table 3:** Reported number of children affected by melamine in China (of 22.4 million patients screened) as of 1 December 2008 (Chinese Ministry of Health 2008).

Status	No.	Percentage of reported cases
Cases reported	294,000	100
Cases hospitalized	51,900	17.6
Hospitalized cases already discharged	51,039	17.4
Hospitalized cases still in serious condition	154	0.05
Cases still in hospital	861	0.3
Deaths	6	0.002
